# Analysis of Peripapillary and Macular Choroidal Thickness in Eyes with Pseudoexfoliative Glaucoma and Fellow Eyes

**DOI:** 10.1155/2020/9634543

**Published:** 2020-06-07

**Authors:** Fan Li, Qingli Shang, Guangxian Tang, Hengli Zhang, Xiaowei Yan, Lihua Ma, Yulei Geng, Qing Zhang

**Affiliations:** ^1^Department of Ophthalmology, Shijiazhuang No. 1 Hospital, Shijiazhuang, Hebei 050000, China; ^2^Department of Ophthalmology, The Second Hospital of Hebei Medical University, Shijiazhuang, Hebei 050000, China

## Abstract

**Purpose:**

To compare differences in peripapillary and macular choroidal thickness in pseudoexfoliative glaucoma (PXG) eyes, nonexfoliative fellow eyes, and normal eyes.

**Methods:**

This case-control study included 37 PXG patients (group A: 37 PXG eyes; group B: 37 nonexfoliative fellow eyes) and 37 sex-, age-, and axial length-matched healthy volunteer eyes (group C). Peripapillary and macular choroidal thickness and volume were measured in all subjects via enhanced-depth imaging-optical coherence tomography.

**Results:**

The average peripapillary (AP) choroidal thickness was (130.10 ± 46.14) *μ*m, (131.43 ± 46.00) *μ*m, and (147.89 ± 53.32) *μ*m; average macular (AM) choroidal thickness was (191.72 ± 68.07) *μ*m, (204.62 ± 69.54) *μ*m, and (215.10 ± 45.40) *μ*m; and average volume was (0.59 ± 0.21) *μ*m^3^, (0.63 ± 0.21) *μ*m^3^, and (0.65 ± 0.14) *μ*m^3^ in groups A, B, and C, respectively. NIP choroidal thickness was significantly lower in groups A and B than in group C (*P* < 0.05). TIM and TOM choroidal thickness and volume were significantly lower in group A than in group C (*P* < 0.05). NIM, SIM, NOM, IOM, AM choroidal thickness and volume, and CSM choroidal thickness were significantly lower in group A than in group B (*P* < 0.05). CSM, TIM, and TOM in group A and TIM, TOM choroidal thickness, and volume in group B were significantly lower than in group C (*P* < 0.05).

**Conclusions:**

NIP choroidal thickness in PXG eyes and nonexfoliative fellow eyes and temporal macular choroidal thickness in PXG eyes were significantly lower than in normal eyes. Macular choroidal thickness (except in temporal regions) was significantly lower in PXG eyes than in nonexfoliative fellow eyes. Changes in peripapillary and macula choroidal thickness further elucidate the choroid's role in PXG development and progression.

## 1. Introduction

Pseudoexfoliative glaucoma (PXG) is a secondary glaucoma caused by pseudoexfoliative syndrome (PEX) and accounts for approximately 25% of all open-angle glaucoma cases [1–3]. PXG is characterized by elevated intraocular pressure (IOP) due to blockage of the trabecular meshwork by pseudoexfoliative materials and pigments. The pseudoexfoliative materials not only deposit on the anterior segment tissues of the eye such as the corneal endothelium, lens surface, and trabecular meshwork but may also have an impact on the posterior segment tissues of the eye, including the posterior ciliary arteries, vorticose veins, and central retinal vessels [4, 5]. Unilateral PEX is not truly unilateral but rather an asymmetrical manifestation in the two eyes. Exfoliating material has been detected around iris blood vessels of non-PEX nonexfoliative fellow eyes through ultrastructural and immunohistochemical methods [6]. A previous study found hemodynamic abnormalities in the posterior ocular vessels of PEX and PXG patients [7]. The choroid is a layer of blood vessels located under the retina that accounts for 70–80% of the blood flow to the eye and has the highest perfusion rate of all the blood vessels in the human body. Because of its critical function in ocular blood flow, the choroid plays an important role in the development and progression of glaucoma [8]. The ocular choroidal blood supply derives mainly from the long and short ciliary arteries and partially from the anterior ciliary artery. PXG eyes have abnormal hemodynamics in the posterior ocular artery with decreased end-diastolic flow velocity in the long and short ciliary arteries and an increased resistance index of the posterior short ciliary artery. pseudoexfoliative materials easily affect small blood vessels but not large blood vessels. These pathological changes can all cause thinning of the choroid [9–12].

Enhanced-depth imaging-optical coherence tomography (EDI-OCT) can provide important information on the velocity of choroidal blood flow. With the application of EDI-OCT, it is possible to capture an image of the full thickness of the choroid in vivo [13]. Previous studies of the choroidal thickness of PXG eyes have reported controversial results. One study found that the peripapillary and macular choroidal thickness of PXG eyes measured with spectral-domain optical coherence tomography (SD-OCT) was decreased compared to that of normal eyes [14]. Another study reported that the peripapillary and macular choroidal thickness measured with SD-OCT in PXG eyes did not differ significantly from that of normal eyes [15]. No studies have compared the peripapillary and macular choroidal thickness and volume of PXG eyes, nonexfoliative fellow eyes, and normal eyes. In addition, whether exfoliating material can cause abnormal ocular blood flow is still not clear. Measurement of choroidal thickness through enhanced-depth imaging-optical coherence tomography (EDI-OCT) can provide important information on the velocity of choroidal blood flow [8]. In this study, using EDI-OCT, we measured the peripapillary and macular choroidal thickness and volume of PXG eyes, nonexfoliative fellow eyes, and normal eyes among the Chinese population and analyzed changes in choroidal thickness. We aimed to determine the effect of the choroid on PXG development and progression.

## 2. Materials and Methods

A total of 37 PXG patients treated in our hospital between December 2014 and December 2019 were recruited for this study. The PXG eyes (37 eyes) were included in group A, and the nonexfoliative fellow eyes (37 eyes) were included in group B. Thirty-seven sex-, age-, and axial length-matched healthy volunteers (37 eyes) were recruited during the same time period and included in group C. The number of local antiglaucoma medications used by the patients in group A was 1.81 ± 1.02 (0–3) (Table 1).

Inclusion criteria were as follows: (1) the eyes in group A met the PXG diagnostic criteria: gray-white pseudoexfoliative material was found at the pupillary margin, iris surface, and anterior lens capsule; the IOP was >21 mmHg; and the patients had glaucomatous optic neuropathy and visual field changes [1]. The eyes in group B were nonexfoliative fellow eyes; ocular slit lamp examination did not detect any gray-white pseudoexfoliative material, the IOP was <21 mmHg, and the eyes had no history of elevated IOP, no glaucomatous optic disc appearance, and no obvious nerve fiber layer defects. The results of standard automatic visual field examinations were normal. (2) The age of the subjects was ≥50 years. (3) Diopter: the equivalent spherical degree was ≤±6.0 D, and the cylindrical degree was ≤±3.0 D.

Exclusion criteria were as follows: (1) patients with other types of glaucoma such as angle-closure glaucoma or secondary glaucoma; (2) patients with a history of ocular surgery or a history of ocular trauma; (3) patients with nonglaucomatous optic neuropathy or visual field defects; (4) patients with active, chronic, or recurrent uveitis; (5) patients with retinal choroidal lesions due to diabetic retinopathy or macular degeneration; (6) patients with systemic diseases such as diabetes or hypertension; and (7) patients who had been or were being treated with systemic antiglaucoma medications. This study was conducted according to the principles set forth in the Declaration of Helsinki and was approved by the ethics committee of Shijiazhuang No. 1 Hospital. All subjects or their guardians signed informed consent forms.

### 2.1. Routine Examinations

All subjects underwent comprehensive eye examinations, including vision tests, slit lamp microscopy, IOP measurement (Goldmann applanation tonometer), axial length measurement, gonioscopy, and fundus and visual field examinations.

### 2.2. OCT Examinations

**Figure 1:**
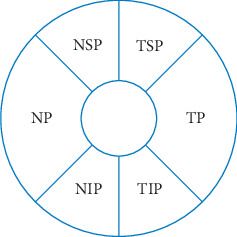
Measurement illustration of peripapillary choroidal thickness at six locations. NP, nasal peripapillary; NSP, nasal superior peripapillary; TSP, temporal superior peripapillary; TP, temporal peripapillary; TIP, temporal inferior peripapillary; NIP, nasal inferior peripapillary.

**Figure 2:**
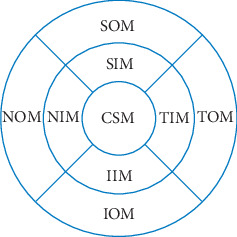
Measurement illustration of macular choroidal thickness at nine locations. CSM, central subﬁeld macula; NIM, nasal inner macula; SIM, superior inner macula; IIM, inferior inner macula; TIM, temporal inner macula; NOM, nasal outer macula; SOM, superior outer macula; IOM, inferior outer macula; TOM, temporal outer macula.

All subjects underwent the SD-OCT (Spectralis HRA + OCT, Heidelberg, Germany) optic disc examination procedure, including a circular scan on EDI mode centered on the optic disc with a diameter of 3.4 mm. The choroidal thickness of six regions of the peripapillary area, including the nasal peripapillary (NP), nasal superior peripapillary (NSP), temporal superior peripapillary (TSP), temporal peripapillary (TP), temporal inferior peripapillary (TIP), and nasal inferior peripapillary (NIP) regions, as well as the average peripapillary (AP) choroidal thickness, was measured (Figure 1). The macular area was scanned using the EDI mode of the SD-OCT macular thickness map (MTP) examination procedure with the following parameters: the wavelength of the light source was 870 nm, the vertical resolution was 5 *μ*m, the lateral resolution was 6 *μ*m, and the scanning was centered on the macula with a volume of 30° × 25°. According to the definition of the Early Treatment of Diabetic Retinopathy Study (ETDRS), the macular region was divided into three concentric circles, specifically, the central region (1 mm in diameter), the inner ring zone (1 to 3 mm), and the outer ring zone (3 to 6 mm). Each ring zone was subdivided into four quadrants by two radiation lines: the upper quadrant (45° to <135°), the nasal quadrant (135° to <225°), the lower quadrant (225° to <315°), and the temporal quadrant (315° to <45°). The choroidal thickness of nine macular regions, namely, the central subﬁeld macula (CSM), the nasal inner macula (NIM), the superior inner macula (SIM), the inferior inner macula (IIM), the temporal inner macula (TIM), the nasal outer macula (NOM), the superior outer macula (SOM), the inferior outer macula (IOM), and the temporal outer macula (TOM), as well as the average macular (AM) choroidal thickness, was measured (Figure 2). On each scanned image, the inscribed segmentation line was labeled on the retinal pigment epithelium (RPE)/Bruch membrane interface to indicate the internal choroidal boundary, and the outer segmentation line was placed on the scleral/choroidal interface to indicate the external choroidal boundary.

### 2.3. Visual Field Examination

The visual fields of all subjects were examined using the SITA-Fast 30–2 examination procedure and a Humphrey 750i visual field analyzer (Carl Zeiss, Germany). The reliability criteria included a fixation loss rate of <20%, a false negative rate of <15%, and a false positive rate of <15%. Individuals who did not meet the criteria were excluded.

### 2.4. Statistical Analysis

The data were analyzed using SPSS 19.0 statistical software (IBM Corporation, Armonk, NY, USA). The measurement data were normally distributed; therefore, the choroidal thickness and volume values of PXG eyes and nonexfoliative fellow eyes were compared using the paired *t*-test. An independent *t*-test was used for choroidal thickness and volume comparisons between PXG eyes and control subjects and between fellow eyes and control subjects.

## 3. Results

### 3.1. Comparison of Peripapillary Choroidal Thickness

**Figure 3:**
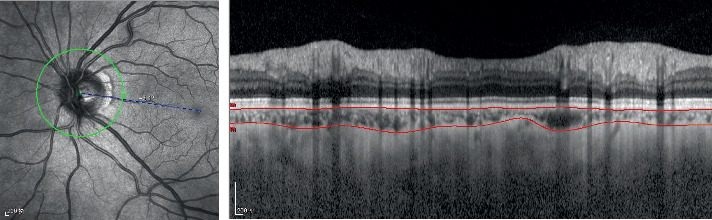
Optical coherence tomographic image (enhanced-depth imaging mode) for measurement of the peripapillary choroidal thickness.

The AP choroidal thickness in groups A, B, and C was (130.10 ± 46.14) *μ*m, (131.43 ± 46.00) *μ*m, and (147.89 ± 53.32) *μ*m, respectively (Figure 3). The NIP choroidal thickness was significantly lower in groups A and B than in group C (*P* < 0.05), while the choroidal thickness of the other regions and the average choroidal thickness in groups A and B did not differ significantly from those in group C (*P* > 0.05). The choroidal thickness of each region and the average choroidal thickness in groups A and B did not differ significantly (*P* > 0.05) (Table 2).

### 3.2. Comparison of Macular Choroidal Thickness and Volume

**Figure 4:**
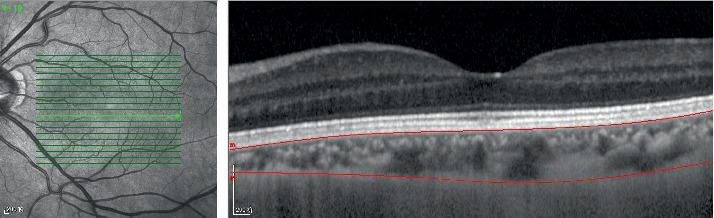
Optical coherence tomographic image (enhanced-depth imaging mode) for measurement of the macular choroidal thickness.

The AM choroidal thickness in groups A, B, and C was (191.72 ± 68.07) *μ*m, (204.62 ± 69.54) *μ*m, and (215.10 ± 45.40) *μ*m, respectively, and the average choroidal volume was (0.59 ± 0.21) *μ*m^3^, (0.63 ± 0.21) *μ*m^3^, and (0.65 ± 0.14) *μ*m^3^, respectively (Figure 4). CSM, NIM, SIM, NOM, IOM, and AM choroidal thickness was significantly lower in group A than in group B (*P* < 0.05), while the choroidal thickness of the other regions did not differ significantly in groups A and B (*P* > 0.05). NIM, SIM, NOM, IOM, and AM choroidal volume was significantly lower in group A than in group B (*P* < 0.05), while the choroidal volumes of the other regions did not differ significantly in groups A and B (*P* > 0.05). CSM, TIM, and TOM choroid thickness and volume were significantly lower in group A than in group C (*P* < 0.05). The choroidal thickness and volume of other macular regions and the AM choroidal thickness and volume in group A did not differ significantly from those in group C (*P* > 0.05). TIM and TOM choroid thickness and volume were significantly lower in group B than in group C (*P* < 0.05). The choroidal thickness and volume of other macular regions and the AM choroidal thickness and volume did not differ significantly in groups B and C (*P* > 0.05) (Table 3).

## 4. Discussion

The pathogenesis of glaucoma is not yet clear. Hemodynamic abnormalities in the optic disc, retina, and choroid may play an important role in the etiology of glaucoma [13]. Galassi et al. [16] found that PXG eyes were more prone to reduced perfusion pressure and abnormal retrobulbar vascular hemodynamics than normal eyes and believed that damaged vascular regulation or deposition of pseudoexfoliative materials in ocular blood vessels was involved in PXG development. Previous studies [17, 18] confirmed that open-angle glaucoma is not associated with significant thinning or thickening of the choroid based on EDI-OCT measurements. Koz et al. [19] found that a significant proportion of PEX patients with normal IOP also had glaucomatous changes and speculated that the optic disc damage in PXG eyes might not be related to IOP. The large range of IOP fluctuations in PXG eyes may be an important factor underlying glaucoma progression, but the impact of pseudoexfoliation and choroidal dysfunction on glaucoma progression cannot be ruled out.

The pathogenesis of glaucoma is closely related to retrobulbar blood flow. The choroidal blood supply around the optic papilla derives from the posterior ciliary artery and the Zinn-Haller arterial ring in the sclera, providing blood for the cribriform plate at the optic nerve head [20, 21]. Abnormal choroidal blood supply can cause glaucomatous optic neuropathy [22], and blood flow resistance is related to the diameter of blood vessels [23]. Jiang et al. [24] showed that the peripapillary choroidal thickness of the normal eye is related to age and axial length, with the top being the thickest and the bottom being the thinnest. The choroidal thickness is reduced by approximately 2 *μ*m with each year of age and by approximately 5 *μ*m with each additional diopter. Ayhan et al. [14] found that, with the exception of the top region, the peripapillary choroidal thickness of PXG eyes was lower than that of normal eyes. Conversely, Ozge [15] et al. reported that the peripapillary choroidal thickness of PXG eyes does not differ significantly from that of normal eyes. We found that the NIP choroids of PXG eyes and nonexfoliative fellow eyes were thinner than those of normal eyes and that the choroidal thickness of the individual peripapillary regions in PXG eyes and nonexfoliative fellow eyes did not differ significantly. Thinning of the peripapillary choroid is of important significance because glaucomatous optic neuropathy occurs in the optic papilla region [25]. The AP choroidal thickness in the PXG eye group, the nonexfoliative fellow eye group, and the normal eye group was (130.10 ± 46.14) *μ*m, (131.43 ± 46.00) *μ*m, and (147.89 ± 53.32) *μ*m, respectively. The distribution of peripapillary choroidal thickness was TSP > NSP > TP > NP > TIP > NIP for both the PXG eyes and nonexfoliative fellow eyes, and the distribution in normal eyes was TSP > NSP > NP > TP > NIP > TIP. Based on these distributions, we speculate that the NIP choroidal thickness is reduced more rapidly than that of other peripapillary regions during PXG progression.

Previous studies of the macular choroidal thickness of PXG eyes have reported controversial results. Ayhan et al. [14] found that the macular central foveal and parafoveal (500 *μ*m and 1500 *μ*m nasal and temporal from the central fovea) choroidal thickness was lower in PXG eyes than in normal eyes. However, Ozge et al. [15] showed that the central foveal and parafoveal (500 *μ*m and 1500 *μ*m nasal and temporal from the central fovea) choroidal thickness did not differ significantly in PXG eyes and normal eyes.

Our study showed that the temporal macular choroid of PXG eyes and nonexfoliative fellow eyes was thinner than that of normal eyes and that macular choroidal thickness and volume (except in the temporal region) were significantly lower in PXG eyes than in nonexfoliative fellow eyes. The AM choroidal thickness of PXG, nonexfoliative fellow eyes, and normal eyes was (191.72 ± 68.07) *μ*m, (204.62 ± 69.54) *μ*m, and (215.10 ± 45.40) *μ*m, respectively. The distribution of choroidal thickness in all three groups was inner macula > outer macula. The distribution of inner macular choroidal thickness in the PXG and nonexfoliative fellow eyes was SIM > TIM > IIM > NIM, and the distribution of outer macular choroidal thickness in these eyes was SOM > IOM > TOM > NOM. In normal eyes, the distribution of inner macular choroidal thickness was TIM > SIM > IIM > NIM, and the distribution of outer macular choroidal thickness was SOM > TOM > IOM > NOM. Therefore, we speculate that the temporal macular choroidal thickness is reduced more rapidly during PXG progression.

Our study further suggests that PEX is a binocular lesion and that nonexfoliative fellow eyes with normal IOP are not truly normal eyes. The choroidal thickness of the nonexfoliative fellow eyes may have already changed prior to observation of the presence of pseudoexfoliative materials under a slit lamp microscope. Our study, together with a previous study [14], confirms that the choroid of some peripapillary and macular regions in PXG eyes is thinner than that in normal eyes, but the specific regions that show thinning are inconsistent. This inconsistency may be due to the use of different choroid divisions and different measurement locations.

This study has some limitations. First, the sample size was relatively small. We set strict inclusion criteria to ensure that the observation group and the control group had matched parameters, such as axial length, and used nonexfoliative fellow eyes as control to reduce interference from individual variations that might affect the results. Second, the choroidal thickness was measured manually due to a lack of automatic measurement software, which may have resulted in some error in our results. Third, the antiglaucoma medications taken by the PXG patients may affect choroidal thickness [26, 27]. Previous studies have shown that *α*_2_ receptor agonists and carbonic anhydrase inhibitors can increase choroidal blood flow [28, 29]. Additionally, the choroid is a highly dynamic vascular tissue, and simple measurement of choroidal thickness cannot sufficiently describe the hemodynamic and physiological changes that occur in ocular diseases. Therefore, assessment of the clinical impact of changes in choroidal thickness on glaucoma requires further investigation in large-scale multicenter studies.

## Figures and Tables

**Table 1:** Baseline characteristics of the study groups are shown in three groups.

	Group A (37 eyes)	Group B (37 eyes)	Group C (37 eyes)	P value		
				A-B^a^	A-C^b^	B-C^b^
Age (year)	72.05 ± 8.53 (50–87)		74.86 ± 7.80 (56–86)		0.158	
Sex (M/F)	19/18		17/20		0.642	
AL (mm)	23.17 ± 0.94	23.13 ± 0.97	23.20 ± 0.82	0.321	0.903	0.750
MD (dB)	−12.87 ± 10.15	−1.14 ± 0.40	−1.15 ± 0.40	0.0001	0.0001	0.982
IOP (mmHg)	36.57 ± 8.85 (26–55)	16.51 ± 2.01 (12–20)	16.16 ± 2.08 (10–20)	0.0001	0.0001	0.462

M, male; F, female; IOP, intraocular pressure; AL, axial length; MD, mean defect. ^a^Paired *t*-test; ^b^independent *t*-test.

**Table 2:** Comparisons of peripapillary choroidal thickness by EDI-OCT in three groups.

Location	Group A	Group B	Group C	P value		
				A-B^a^	A-C^b^	B-C^b^
NP	131.43 ± 49.78	131.24 ± 50.62	152.03 ± 56.15	0.980	0.092	0.099
NSP	144.92 ± 49.95	143.68 ± 49.84	165.95 ± 62.62	0.872	0.109	0.095
TSP	146.54 ± 52.82	152.30 ± 59.14	167.95 ± 60.01	0.490	0.097	0.262
TP	134.57 ± 59.96	140.38 ± 59.57	146.24 ± 60.41	0.443	0.387	0.675
TIP	112.97 ± 41.30	110.84 ± 38.88	124.68 ± 48.26	0.668	0.251	0.179
NIP	109.03 ± 35.88	108.78 ± 39.72	129.70 ± 44.44	0.865	0.033^※^	0.036^※^
AP	130.10 ± 46.14	131.43 ± 46.00	147.89 ± 53.32	0.842	0.115	0.155

NP, nasal peripapillary; NSP, nasal superior peripapillary; TSP, temporal superior peripapillary; TP, temporal peripapillary; TIP, temporal inferior peripapillary; NIP, nasal inferior peripapillary; AP, average peripapillary. Data are expressed as means ± standard deviation. ^※^Significant; ^a^paired *t*-test; ^b^independent *t*-test.

**Table 3:** Comparisons of macular choroidal thickness by EDI-OCT in three groups.

Location		Group A	Group B	Group C	P value		
					A-B^a^	A-C^b^	B-C^b^
CSM	TH, *μ*m	201.84 ± 71.33	216.70 ± 79.13	232.92 ± 50.23	0.047^※^	0.034^※^	0.297
	*V*, *μ*m^3^	0.16 ± 0.06	0.16 ± 0.07	0.18 ± 0.04	0.409	0.038^※^	0.164
NIM	TH, *μ*m	184.92 ± 73.29	206.54 ± 79.43	206.89 ± 55.31	0.002^※^	0.150	0.982
	*V*, *μ*m^3^	0.29 ± 0.12	0.32 ± 0.13	0.33 ± 0.09	0.024^※^	0.146	0.779
SIM	TH, *μ*m	208.86 ± 74.44	224.95 ± 79.13	232.70 ± 46.78	0.034^※^	0.104	0.610
	*V*, *μ*m^3^	0.33 ± 0.12	0.35 ± 0.12	0.37 ± 0.07	0.037^※^	0.109	0.603
TIM	TH, *μ*m	199.32 ± 72.49	206.65 ± 68.97	234.92 ± 47.48	0.343	0.015^※^	0.044^※^
	*V*, *μ*m^3^	0.31 ± 0.12	0.32 ± 0.11	0.37 ± 0.07	0.322	0.014^※^	0.042^※^
IIM	TH, *μ*m	195.24 ± 76.46	205.35 ± 75.75	215.76 ± 50.95	0.117	0.179	0.491
	*V*, *μ*m^3^	0.31 ± 0.12	0.32 ± 0.12	0.34 ± 0.08	0.096	0.159	0.464
NOM	TH, *μ*m	157.97 ± 68.92	172.57 ± 71.53	170.16 ± 60.89	0.022^※^	0.423	0.877
	*V*, *μ*m^3^	0.84 ± 0.36	0.92 ± 0.38	0.90 ± 0.32	0.020^※^	0.427	0.864
SOM	TH, *μ*m	208.03 ± 67.78	219.22 ± 70.41	226.84 ± 47.45	0.070	0.171	0.587
	*V*, *μ*m^3^	1.10 ± 0.36	1.16 ± 0.38	1.20 ± 0.25	0.094	0.181	0.594
TOM	TH, *μ*m	182.05 ± 60.20	189.24 ± 58.35	213.16 ± 43.25	0.262	0.013^※^	0.049^※^
	*V*, *μ*m^3^	0.97 ± 0.32	1.00 ± 0.31	1.13 ± 0.23	0.270	0.013^※^	0.047^※^
IOM	TH, *μ*m	187.24 ± 72.13	200.35 ± 72.10	202.57 ± 54.13	0.011^※^	0.305	0.882
	*V*, *μ*m^3^	0.99 ± 0.38	1.06 ± 0.38	1.07 ± 0.29	0.011^※^	0.308	0.885
AM	TH, *μ*m	191.72 ± 68.07	204.62 ± 69.54	215.10 ± 45.40	0.018^※^	0.087	0.445
	*V*, *μ*m^3^	0.59 ± 0.21	0.63 ± 0.21	0.65 ± 0.14	0.019^※^	0.114	0.479

CSM, central subfield macula; NIM, nasal inner macula; SIM, superior inner macula; IIM, inferior inner macula; TIM, temporal inner macula; NOM, nasal outer macula; SOM, superior outer macula; IOM, inferior outer macula; TOM, temporal outer macula; AM, average macula; TH, thickness; *V*, volume. Data are expressed as means ± standard deviation. ^※^Significant; ^a^paired *t*-test; ^b^independent *t*-test.

## Data Availability

The research data used to support the findings of this study are available from the corresponding author upon request.
